# Are Assistant Matrons Unbusinesslike?

**Published:** 1916-02-19

**Authors:** 


*To the Editor of* THE HOSPITAL.

### Are Assistant Matrons Unbusinesslike?

*To the Editor of* THE HOSPITAL.

SIR,—The secretary of a large London hospital noted the other day that in reply to an advertisement for an assistant matron a large number of answers were received. The advertisement requested each candidate to apply for an official form of application. This form was drawn up with extreme care and after long deliberation. I have seen a specimen of the form, and it is quite intelligible and clear. The idea of the form is to save the selection committee much time and labour in going through a large number of applications. The form in itself summarises and condenses the particulars that each applicant is requested to furnish. Yet it seems that quite a large proportion of the ladies who applied for the post have not only filled in the forms in a careless and unbusinesslike way, but in some instances applicants have actually crossed out with their own pens some of the printed wording upon the forms. A number of the applicants have sent in the forms in one envelope and their testimonials in another. Other applicants have written separate and futile letters, asking for particulars that have already been given either in the advertisement or upon the form. Very few of the applicants have taken the trouble to attach the copies of their testimonials to the form. Many of the copies of testimonials sent in are undated. In these days, when women are doing much useful work, it seems a pity that they cannot acquire more businesslike and thoughtful methods.—I am, yours, etc., SUPERINTENDENT.

